# His-Ala-Phe-Lys peptide from *Burkholderia arboris* possesses antifungal activity

**DOI:** 10.3389/fmicb.2022.1071530

**Published:** 2022-12-06

**Authors:** Huajie Zhu, Cuihong Xu, Yicun Chen, Yan Liang

**Affiliations:** ^1^State Key Laboratory for Managing Biotic and Chemical Threats to the Quality and Safety of Agro-products, Institute of Biotechnology, Zhejiang University, Hangzhou, China; ^2^Research Institute of Subtropical Forestry, Chinese Academy of Forestry, Hangzhou, China

**Keywords:** *Burkholderia arboris*, antifungal ability, Tn5, untargeted metabolomics, peptide

## Abstract

*Burkholderia arboris*, which belongs to the *Burkholderia cepacia* complex, has been shown to possess antifungal activity against several plant fungal pathogens; however, the antifungal compounds are yet to be identified. Here, we identified the antifungal compounds produced by *B. arboris* using genetic and metabolomic approaches. We generated a Tn5 transposon mutation library of 3,000 *B. arboris* mutants and isolated three mutants with reduced antifungal activity against the plant fungal pathogen *Fusarium oxysporum*. Among the mutants, the M464 mutant exhibited the weakest antifungal activity. In the M464 genome, the transposon was inserted into the *cobA* gene, encoding uroporphyrin-III methyltransferase. Deletion of the *cobA* gene also resulted in reduced antifungal activity, indicating that the *cobA* gene contributed to the antifungal activity of *B. arboris*. Furthermore, a comparison of the differential metabolites between wild type *B. arboris* and the ∆*cobA* mutant showed a significantly decreased level of tetrapeptide His-Ala-Phe-Lys (Hafk) in the ∆*cobA* mutant. Therefore, a Hafk peptide with D-amino acid residues was synthesized and its antifungal activity was evaluated. Notably, the Hafk peptide displayed significant antifungal activity against *F. oxysporum* and *Botrytis cinerea*, two plant pathogens that cause destructive fungal diseases. Overall, a novel antifungal compound (Hafk) that can be used for the biocontrol of fungal diseases in plants was identified in *B. arboris*.

## Introduction

Protection against crop losses caused by pathogens, especially fungi, is important to ensure sustainable crop production to meet the demand of the growing global population (Nazarov et al., 2020). Although chemical control using synthetic fungicides is effective in reducing crop loss caused by fungal diseases (Russell, 2005; Goldenhar and Hausbeck, 2019), there has been an increased concern on the potential environmental (Latin and Ou, 2018; Fraaije et al., 2020; Krichilsky et al., 2021) and health risks of indiscriminate fungicides use, as well as the rising threat of fungicide resistance in plant pathogens (Canal-Raffin et al., 2008; van den Bosch and Gilligan, 2008). Therefore, biological control using microbes or microbial compounds has been suggested as a sustainable alternative for plant protection against fungal diseases (Joo and Hussein, 2022). Since *Bacillus* was used as a biocontrol agent in 1940, large efforts have been made to identify effective biocontrol bacteria, most of which belong to the *Bacillus* and *Pseudomonas* genera (Poveda et al., 2021). Biocontrol bacteria antagonize pathogens mainly by producing bioactive compounds, including antibiotics, siderophores, peptides, lipopeptides, polyketides, bacteriocins, and enzymes (Mercado-Blanco and Bakker, 2007; Daungfu et al., 2019; Chen et al., 2020). Antagonistic compounds are primarily produced by non-ribosomal peptide synthase (NRPS) and polyketide synthases (PKS). NRPS are multi-domain and multi-enzymatic mega-synthases responsible for the synthesis of non-ribosomal peptides, sometimes with modifications, such as oxidation, acylation, methylation, or glycosylation (Marahiel, 2009; Thomson and Dennis, 2012). PKS is involved in the synthesis of polyketides, similar to fatty acid synthase (Staunton and Weissman, 2001; Hertweck, 2009). Among these antagonistic compounds, antimicrobe peptides, including lipopeptides, glycans, and cyclic peptides, have received substantial attention owing to their ecofriendly characteristics (Fira et al., 2018). The genome sequence of *B. subtilis* revealed that approximately 5% of its genome is dedicated to the production of antimicrobe compounds (Caulier et al., 2019; Tran et al., 2022). Among 2,700 antimicrobial peptides in Antimicrobial Peptide Database, 1,000 of them have antifungal activity, and most are produced by *Bacillus*, *Paenibacillus*, *Pseudomonas*, and *Aspergillus* (Li et al., 2021). Antifungal peptides are usually referred to as peptides containing 10–100 amino acid residues (Zhang et al., 2020); however, more and more short chain polypeptides have been found to process antifungal ability. His (2-aryl)-Trp-Arg has been reported to antagonize *Cryptococcus neoformans* (Sharma et al., 2021). In *B. subtilis*, 57 peptides with less than five amino acids were found to have antifungal activity, among which two tetrapeptides AWYW and HWWY strongly suppressed the growth of *Phytophthora capsici* and *Penicillin chrysogenum* (Qiao et al., 2020).

*Burkholderia* is a genus comprising over 60 species of gram-negative bacteria, and are widely present in soil around the rhizosphere zone, water, plants, and animals (Mahenthiralingam et al., 2008). *Burkholderia* was firstly defined in 1942 by Burkholder and initially named *Pseudomonas caryophyll* until 1992 when seven species with similar genetic characters were transferred from the *Pseudomonas* genus to establish the genus *Burkholderia* (Burkholder, 1942; Yabuuchi et al., 1992). *Burkholderia* species have been utilized as natural sources of biotechnological agents in agricultural production owing to their ability to produce several biomolecules, including antibiotics; extracellular hydrolytic enzymes, such as chitinase, glucanase, and protease (Elshafie and Camele, 2021); and bioactive secondary metabolites, such as alkaloids, lipopeptides, and polypeptides (El-Banna and Winkelmann, 1998; Sultan et al., 2008). *Burkholderia cepacia* complex (BCC) is a group of antibiotic resistant bacteria, including *Burkholderia arboris*, capable of producing bioactive compounds *via* NRPS and PKS (Thomson and Dennis, 2012). *Burkholderia arboris* has been widely isolated from the rhizosphere zone of the soil, and has been shown to possess antifungal activity (Barrera-Galicia et al., 2021); however, the antifungal compound is yet to be identified.

Here, we identified the antifungal compound in *B. arboris* using molecular and metabolomic techniques and evaluated the antifungal activity against selected plant fungi. It is anticipated that the antifungal agent identified in *B. arboris* would be effective as a biocontrol agent against phytopathogenic fungi causing Fusarium wilt and gray mold disease.

## Materials and methods

### Strains and culture conditions

The bacterial and fungal strains used in this study are listed in Supplementary Table 1. *Burkholderia arboris* 1 (CGMCC No.16905), a strain isolated from *Vernicia fordii* (tung oil tree) in China, *Botrytis cinerea* B05.10, and *Fusarium oxysporum* f. sp. *lycopersici* were used in this study. *Burkholderia arboris* was cultured in nutrient agar (NA) medium at 28°C (Edwards et al., 1965), and *F. oxysporum* and *B. cinerea* were cultured in potato dextrose agar (PDA) medium at 28°C (Robbins, 1924). *Escherichia coli* were cultured in Luria-Bertani (LB) medium at 37°C (Elder, 1983). Kanamycin (Kan) was added to the medium at a final concentration of 50 μg/ml when screening for clones positive for *E. coli*, and at 500 μg/ml when screening for clones positive for *B. arboris*.

### Construction of a Tn5 transposon mutant library

The Tn5 transposon mutant library was constructed as previously described (He et al., 2021). Briefly, a fragment of the kanamycin resistance gene (*Kan*) was amplified from plasmid pBBR1MCS-2 by PCR using primers MEKANA-F/R containing Tn5 mosaic end (ME) sequences. The PCR product was ligated into the pCE2 vector using a TA/Blunt-Zero Cloning Kit (Vazyme Biotech, Nanjing, China). After confirmation by sequencing, the Kan-ME fragment was transformed into *B. arboris* using an EZ-Tn5™ Transposase Kit (Lucigen, Middleton, WI, United States). Putative mutants were selected using 500 μg/ml kanamycin.

### Plasmid construction and *cobA* mutant generation

The plasmids used in this study are listed in Supplementary Table 1. The primer sequences used for plasmid construction are listed in Supplementary Table 2. Genomic DNA of *B. arboris* was extracted using a TIANamp Bacteria DNA Kit (Tiangen Biotech, Beijing, China). To knockout *cobA*, a fragment of the *cobA* gene in *B. arboris* genome was amplified by PCR. The PCR product was digested with *Bam*HI and *Eco*RI restriction enzymes and then ligated into the suicide plasmid pJP5603 (Riedel et al., 2013), which was digested with the same enzymes. The ligation product was transferred into *E. coli* S17-1 λpir cells by heat shock. Positive clones were selected with 50 mg/ml kanamycin resistance and verified by sequencing the *cobA* insertion in the pJP5603-*cobA* plasmid. The resulting pJP5603-*cobA* plasmid was transferred into *B. arboris* competent cells with electroporation. Putative mutants were selected with 500 mg/ml kanamycin and verified by PCR with primers from 16S rDNA of *B. arboris* and the conserved R6K origin of replication (Riedel et al., 2013). The transcript levels of *cobA* in mutants were determined by qRT-PCR.

### Identification of Tn5 insertion sites in *Burkholderia arboris* genome

Tn5 insertion sites were identified using thermal asymmetric interlaced PCR (TAIL-PCR; Liu and Whittier, 1995). Arbitrary degenerated primers served as forward primers for all three rounds of PCR, and specific primers, including SP1, SP2, and SP3, were used as the reverse primers for primary, secondary, and tertiary rounds of PCR, respectively. The primer sequences are listed in Supplementary Table 2. The primary-round PCR product was 100-fold diluted and used as a DNA template for the secondary PCR reaction, which was 100-fold diluted and used as the template for the tertiary PCR reaction. Cycling conditions were as previously described.

### Growth curve assay

Growth curves were generated as previously described (Xian et al., 2020). Bacterial suspension was centrifuged at 5,000 rpm for 5 min after overnight culture at 28°C. The pellet was resuspended in ddH_2_O and adjusted to an OD_600_ of 0.005 using fresh medium. Absorbance at OD_600_ was measured with a microplate spectrophotometer (Multiskan™ FC, Thermo Fisher Scientific, Waltham, MA, United States) every 30 min for 36 h to obtain the growth curves. The growth rate, the doubling time and the lagging time were calculated.

### Swimming ability assay

Swimming ability was determined as previously described (Bahar et al., 2009). Bacterial strains were cultured overnight at 28°C until OD_600_ reached 0.6. Thereafter, the bacteria suspension (2 μl) was point-inoculated on the center of a plate containing semi-solid LB broth with 0.3% agar, and the diameter of the swimming zone was measured 2 days after inoculation.

### Biofilm formation assay

Biofilm formation assay was conducted using the crystal violet staining method (Holmberg et al., 2009). Bacterial strains were cultured overnight until the OD_600_ reached 0.5, and then transferred to 96-well cell plate at 100 μl per well and cultured at 28°C for 72 h without shaking. Thereafter, the wells were washed with sterile water twice and stained with 125 μl 0.1% crystal violet (w/v) for 30 min to quantitatively measure the attached biofilms. After removing the dye, wells were thoroughly dried at 37°C, and 1% sodium dodecyl sulfate (SDS) was added to dissolve the biofilm at 200 μl per well. The quantity of biofilm formed was determined at an absorbance of 570 nm using a Multiskan™ FC microplate spectrophotometer (Thermo Fisher Scientific).

### Preparation of crude extracts from *Burkholderia arboris* suspension

Bacterial culture (300 ml) was centrifuged at 4,000 rpm for 20 min after culturing overnight, and the supernatant was mixed with an equal volume of ethyl acetate. The solution from the organic phase was transferred to a conical flask containing anhydrous Na_2_SO_4_ to remove residual moisture, and was finally filtered through a triangular funnel with cotton. Thereafter, the organic phase filtrate was transferred to a pre-weighed collecting bottle, and the ethyl acetate was evaporated with an IKA® RV 10 digital rotary evaporator at 42°C, 80 rpm under control of IKA® MVP 10 basic compact vacuum pump (IKA, Staufen, Germany). The crude extract was dissolved in 1 ml ethyl acetate.

### Determination of antifungal activity of *Burkholderia arboris*

For determination of antifungal activity of *B. arboris* bacteria, fungal mycelial disks and fungal spore suspension were used as described previously with slight modifications (Kumar et al., 2018). Fungal disks and *B. arboris* were co-cultivated on PDA medium with a distance of 30 mm from each other. The inhibitory zone was measured 6 days after co-cultivation. To prepare the fungal spore suspension, the fungal mycelia and spores on the agar plate were washed off with sterile ddH_2_O and then filtered through two layers of sterile gauzes. The concentration of spore suspension was determined with a hemocytometer. For determination of antifungal activity of *B. arboris* extracts, fungal spore suspension (1.0 × 10^5^ CFU/ml, 500 μl) was evenly spread on PDA medium, and then 8 mm round holes were removed from the solid medium using a sterile punch, followed by the addition of 20 μl of melting PDA medium to each hole to seal the bottom. After cooling, 150 μl of crude *B. arboris* extract was added to the holes, and the diameters of the inhibition zones were measured 6 days later.

### Determination of antifungal activity of Hafk peptide and lysophosphatidylethanolamine

Hafk peptides with D-amino acid residues were synthesized by ChenPeptide Biotech (Nanjing, China). LPE (CAS # 53862–35-4) was purchased from Aladdin Ltd. (Shanghai, China). The antifungal activity of Hafk peptides and LPE was determined as described previously with slight modifications (Shi et al., 2010). Briefly, a stock solution of the Hafk peptides (100 mM) and LPE (100 mM) was prepared using ddH_2_O and ethyl acetate, respectively. PDA medium was added to a 12-well cell plate at 2 ml per well, and then 50 μl of different concentrations of the peptide or LPE solution were evenly spread on the surface of the medium. Thereafter, fungal mycelial disks were placed at the center of the dried medium, and the diameters of fungal mycelial growth were measured 3 days later.

### Untargeted metabolomic analysis and identification of Hafk peptide

Untargeted metabolomic analysis of the bacterial sample was performed at Metware Biotechnology Co., Ltd.^1^ as previously described (Oh et al., 2021). Bacterial samples were collected by centrifugation, and then a 500 μl solution (methanol: water = 4: 1, v/v) containing an internal standard was added to each bacterial sample and vortexed for 3 min. Samples were placed in liquid nitrogen for 5 min, dried on ice for 5 min, thawed on ice, and vortexed for 2 min. This freeze–thaw cycle was repeated thrice. Then they were centrifuged at 12,000 rpm for 10 min at 4°C, and 300 μl of supernatant was collected and kept at −20°C for 30 min, followed by further centrifugation at 12,000 rpm for 3 min at 4°C. Thereafter, 200 μl aliquot of the supernatant was collected for UPLC-QTOF/MS analysis. The analytical conditions were as follows: column, Waters ACQUITY UPLC HSS T3 C18 (1.8 μm, 2.1 mm × 100 mm); column temperature, 4°C; flow rate, 0.4 ml/min; injection volume, 2 μl; solvent system, water (0.1% formic acid), and acetonitrile (0.1% formic acid); gradient program, 95: 5 V/V at 0 min, 10: 90 V/V at 11.0 min, 10: 90 V/V at 12.0 min, 95: 5 V/V at 12.1 min, and 95:5 V/V at 14.0 min. Mass spectrometric analysis was performed using a quadrupole time-of-flight mass spectrometer in the positive and negative ion modes. The gas flow rate was set at 8 L/min; gas and sheath temperatures were 325°C; the fragmetor was 135 V; and the nebulizer was 40 V. The ESI voltage was 2,500 V in the positive-ion mode and 1,500 V in the negative-ion mode.

The original data file acquired by UPLC-QTOF/MS was converted into the mzML format using ProteoWizard software. Peak extraction, alignment, and retention time corrections were performed using the XCMS program. The “SVR” method was used to correct the peak area. Peaks with detection rate lower than 50% in each group samples were discarded. Subsequently, metabolic identification information was obtained by searching the laboratory’s self-built database, integrated public database, AI database, and metDNA. Differential metabolites between wild type and mutant *B. arboris* were determined at variable importance in projection (VIP) ≥ 1, *p* < 0.05 (Student’s *t*-test), and absolute log_2_ fold change (FC) ≥ 1.0. VIP values extracted from the OPLS-DA results, which also contained score plots and permutation plots, were generated using MetaboAnalystR package in R software. The data were log transformed (log_2_) and mean centered before OPLS-DA. A permutation test (200 permutations) was performed to avoid overfitting.

Hafk peptide in the bacterial supernatant was identified by LC–MS analysis. The bacterial supernatant (100 ml) was freeze-dried, dissolved in 4 ml 40% acetonitrile, and then subjected to LC–MS (6400 Series Triple Quadrupole LC–MS by Agilent). The standard sample is the synthesized Hafk peptide (Purity: 81.98%).

## Results

### Generation of *Burkholderia arboris* M464 mutant with reduced antifungal activity

**Figure 1:**
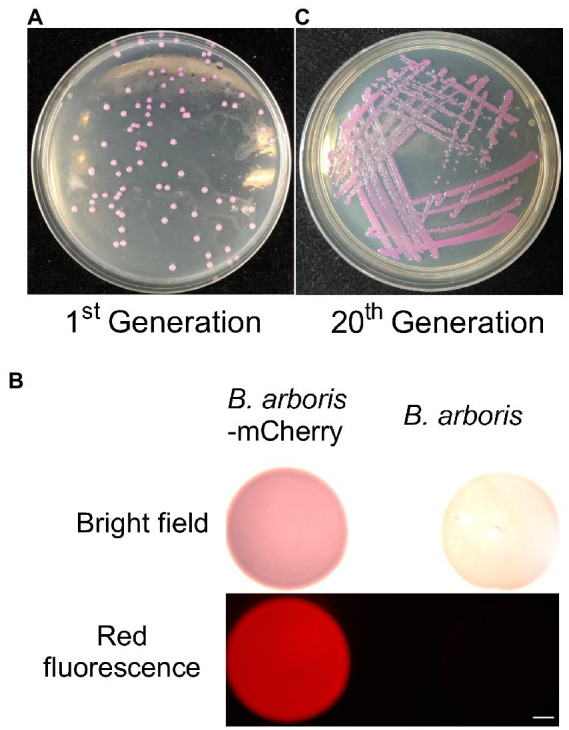
Kanamycin can be used as selection antibiotic for *Burkholderia arboris*. **(A)** Generation of *B. arboris-*mCherry using kanamycin resistance. Plasmids containing mCherry and kanamycin resistance genes were transferred to *B. arboris*, and positive clones were selected on the NA agar plate (90 mm) with 500 μg/ml of kanamycin 48 h after cultivation at 28°C. **(B)** *B. arboris-*mCherry colonies under fluorescent microscopy. Bars, 1,000 mm. **(C)** The morphology of *B. arboris*-mCherry colonies after 20 generations subculturing.

**Figure 2:**
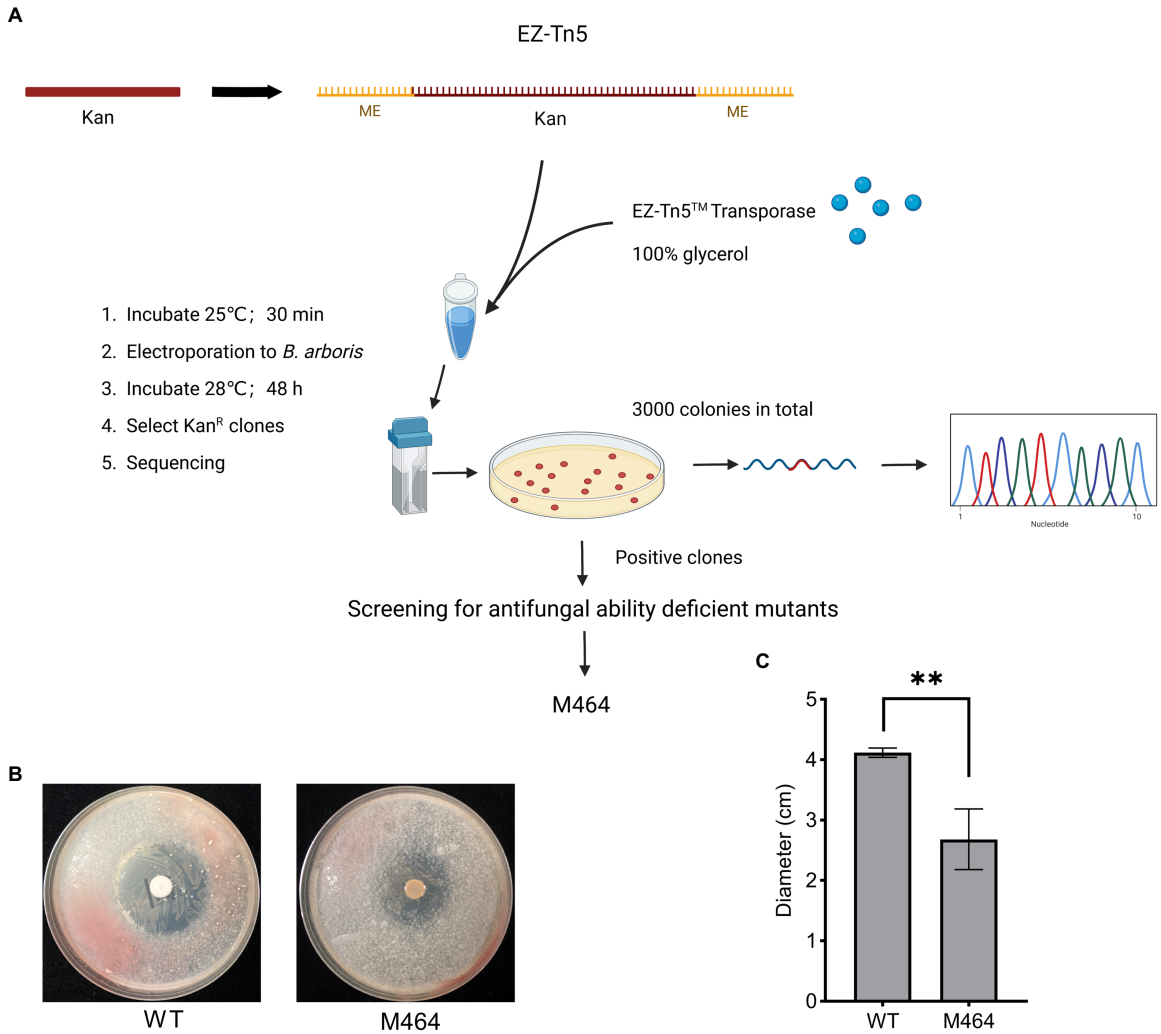
Isolation of *Burkholderia arboris* M464 mutant with reduced antifungal activity. **(A)** Diagram of construction of *B. arboris* Tn5 insertion mutants. **(B)** Confrontation plate experiment of the antifungal activity of *B. arboris* against *Fusarium oxysporum*. Bacterial solution (10 μl at OD_600_ of 1.0) from wild type (WT) and M464 mutant were dropped in the center of 90 mm PDA plates, and fungi (1.0 × 10^5^ spores/ml, 500 μl) were evenly sprayed on each plate 48 h after the cultivation of *B. arboris*. Plates were incubated at 28°C and images were taken 6 days after the fungal spray. **(C)** The M464 mutant showed reduced antifungal activity, as evidenced by the smaller diameter of the inhibition zone against *F. oxysporum*. Data are presented as mean ± SD (*n* = 3). Asterisks indicate significant differences between the wild type and M464 (^**^p < 0.01, Student’s *t*-test). The experiments were repeated twice, and similar results were obtained.

*Burkholderia* spp. are highly resistant to several antibiotics. Therefore, the sensitivity of *B. arboris* to common antibiotics was examined to facilitate genetic analysis of the species. The growth of *B. arboris* was suppressed by 500 μg/ml of Kan, 10-fold higher than the requirement for most bacteria; in contrast, streptomycin (Strep), tetracyclines (Tet), spectinomycin (Spe), and chloroamphenicol (Chl) did not inhibit the growth of *B. arboris* (Supplementary Figure 1). Subsequently, plasmids carrying Kan-resistant and mCherry fluorescent genes were transferred into competent *B. arboris* cells using electroporation (Supplementary Figure 2). Positive clones were selected using NA media containing 500 μg/ml of Kan, and all selected clones displayed pink color under bright field and fluorescent conditions (Figure 1). The pink color did not decrease after 20 generations of subculture. Overall, these results indicated that 500 μg/ml Kan can be used as a selective antibiotic for *B. arboris*. Transposon random mutagenesis technology was used to generate *B. arboris* mutant library. The *Kan* gene fragment (795 bp) was amplified from a plasmid (Supplementary Figure 3) by PCR, ligated to adapters containing a Tn5 mosaic end (ME, 19 bp), and the resulting *Kan-*ME fragments (833 bp) were introduced into *B. arboris* along with transposases (Figure 2A). Overall, 3,000 putative transposon insertion mutants were isolated from Kan-resistant plates.

A plate confrontation experiment was performed to identify *B. arboris* mutants with weak antifungal activity against *Fusarium oxysporum*. Wild type *B. arboris* exhibited high antagonistic activity against *F. oxysporum* (Supplementary Figure 4), whereas three out of 3,000 putative transposon insertion mutants showed reduced antagonistic activity (Supplementary Figure 5). Among the three, M464, with a little yellow color, displayed the weakest inhibitory effect against *F. oxysporum*, as evidenced by the small diameters of the inhibition zones (Figures 2B,C); therefore, it was selected for further analysis.

### *cobA* gene was mutated in M464 mutant

**Figure 3:**
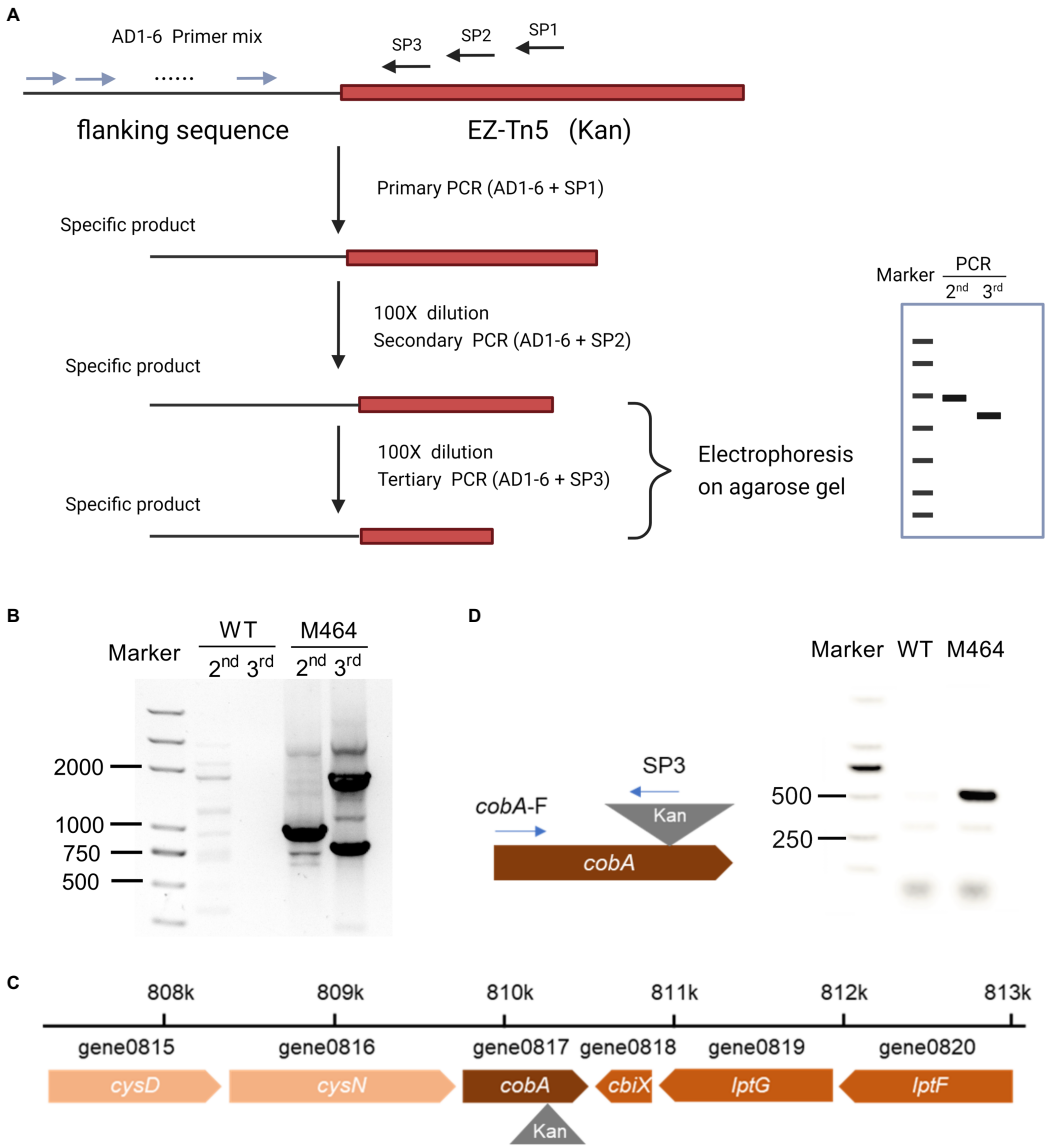
The *cobA* gene was mutated in M464 mutant of *Burkholderia arboris*. **(A)** Diagram of thermal asymmetric interlaced PCR (TAIL-PCR). **(B)** Gel image of TAIL-PCR products. The second and third round of PCR products from wild type (WT) and M464 mutants were run on agarose gel electrophoresis. **(C)** Diagram of the insertion site of the kanamycin resistance gene (*Kan*) in the M464 mutant. The *cobA* gene was mutated in the M464 *B*. *arboris* mutant. **(D)** Fragments of PCR products were detected on agarose gel with specific primers from *cobA* and *Kan* sequences.

The *Kan* fragment from the M464 mutant was amplified by PCR, and the resulting PCR product was electrophoresed on an agarose gel. As expected, 833 bp of PCR fragments were observed in the gel (Supplementary Figure 6), confirming that the *Kan* sequence were inserted into the M464 genome. Thermal asymmetrical interlaced PCR (TAIL-PCR) was performed to amplify the flanking sequence adjacent to the border of *Kan* gene to identify the insertion site of *Kan* sequence in the M464 genome. Three specific primers with high annealing temperatures were designed from the *Kan* gene: SP1, SP2, and SP3. TAIL-PCR was performed using nested specific primers in three subsequent rounds of reactions, together with six arbitrary degenerate (AD) primers (Figure 3A). A specific band of approximately 1,000 bp was obtained in the secondary rounds of PCR with the SP2 primer when the M464 template was amplified, but not the wild type *B. arboris* genomic DNA (Figure 3B). Two bands were obtained during the tertiary rounds of PCR using the SP3 primer (Figure 3B). Since the PCR products from the tertiary rounds of PCR are supposed to be smaller than those from the secondary round, we purified the small band (around 800 bp) and sequenced it using the primer SP3. After alignment with the *B. arboris* genome sequence using the nucleotide Basic Local Alignment Search Tool (BLAST), we found that *Kan* sequence was inserted in the *cobA* gene in *B. arboris* chromosome (Figure 3C). Subsequently, the insertion site of *Kan* was confirmed by PCR using specific primers (Figure 3D). Overall, these results suggest that the *cobA* gene is mutated in M464 mutants.

**Figure 4:**
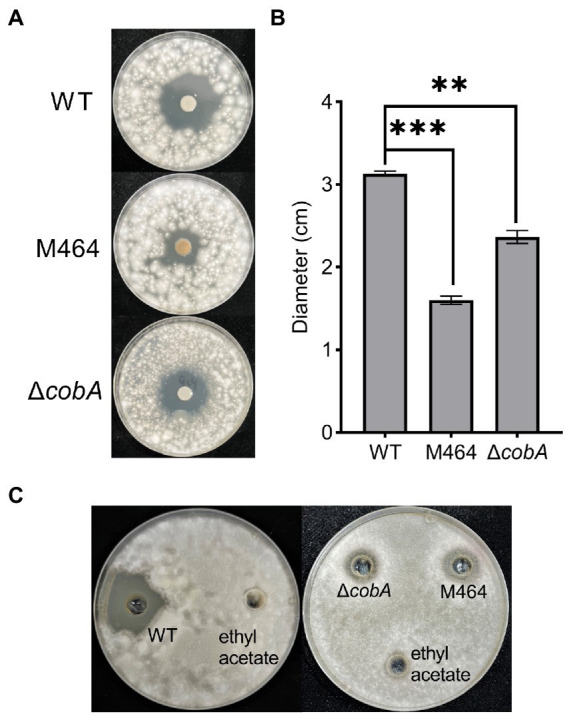
The Δ*cobA* mutant exhibited reduced antifungal activity. **(A)** Confrontation plate experiment of the antifungal activity of *Burkholderia arboris* against *Fusarium oxysporum*. Bacterial solutions (10 μl at OD_600_ of 1.0) from wild type (WT), Δ*cobA*, and M464 mutant were dropped in the center of the 90 mm PDA plate, and fungi (1.0 × 10^5^ spores/ml, 500 μl) were evenly sprayed on each plate 48 h after the cultivation of *B. arboris*. Plates were incubated at 28°C and images were taken 6 days after the fungal spray. **(B)** The Δ*cobA* mutant showed reduced antifungal activity, as evidenced by the small diameter of the inhibition zone. Data are presented mean ± SD (*n* = 3). Asterisks indicate significant differences between the wild type and mutants (^**^p < 0.01, ^***^p < 0.001, Student’s *t*-test). The experiments were repeated twice, and similar results were obtained. **(C)** Crude extract of *B.arboris* inhibits the growth of *F. oxysporum*. Crude metabolites were extracted from the wild-type (WT), Δ*cobA*, and M464 mutants and dissolved in ethyl acetate. The antifungal ability against *F*. *oxysporum* was determined on the PDA plates (90 mm).

A *cobA* knockdown mutant was generated by site-specific mutagenesis to verify that *cobA* is important for the antifungal activity of *B. arboris* against *F. oxysporum*. To achieve this, *cobA* sequence was amplified from the *B. arboris* genome and ligated to the enzyme-digested mobilizable suicide plasmid pJP5603. Knockdown mutants were generated by homologous recombination and screened for resistance to high concentrations of Kan. Once the ∆*cobA* mutant had been verified, a confrontation plate test was performed to evaluate the antifungal activity of ∆*cobA* against *F. oxysporum*. The ∆*cobA* mutants showed significantly lower antifungal activity against *F*. *oxysporum* compared to the wild type, but higher than that of the M464 mutant (Figures 4A,B). Additionally, unlike M464, ∆*cobA* mutants did not show a yellow color, probably because of other mutations in M464. Nevertheless, deletion of *cobA* impaired the antifungal activity of *B. arboris*.

### Mutation of *cobA* gene suppresses the production of antifungal compound

Furthermore, the potential mechanisms by which *cobA* mutation suppressed the antifungal activity of *B. arboris* were examined. First, we determined whether the *cobA* mutation affected the growth of *B. arboris* by comparing the growth curves of the wild type and *ΔcobA* mutant for 36 h, and found that the growth of *ΔcobA* mutant, including the doubling time and lagging time, had no substantial differences from that of the wild type (Supplementary Figure 7 A; Supplementary Table 3). Additionally, biofilm formation assay was performed, and it was observed that *cobA* mutation did not alter biofilm formation by *B. arboris* (Supplementary Figure 7 B). Moreover, swimming ability assay showed that the *ΔcobA* mutant had significantly higher swimming motility than wild type *B. arboris* (Supplementary Figure 7 C). Since *ΔcobA* mutant displayed reduced antifungal activity, it is unlikely that the increased swimming motility of the mutant was the major cause of the impaired antifungal activity. Overall, these results suggest that *cobA* mutation did not affect bacterial growth and physiological characteristics. Therefore, we hypothesized that the *cobA* may be involved in the production of antifungal compounds by *B. arboris*. To verify this hypothesis, the antifungal activities of metabolic crude extracts of the wild type and mutant *B*. *arboris* were evaluated. The crude extract from wild-type *B. arboris* significantly inhibited the growth of *F. oxysporum*, whereas that of *ΔcobA* mutant did not (Figure 4C), confirming that *cobA* is important for the production of antifungal compounds.

### Differential metabolites between the wild type and ∆*cobA* mutant

Untargeted metabolomics was performed to identify the antifungal compounds in *B. arboris*, using UPLC-QTOF/MS, and differential metabolites between *B. arboris* and the *ΔcobA* mutant were identified (Supplementary Table 4). Compared with wild type *B. arboris*, 17 compounds were identified in the *ΔcobA* mutant, among which three were downregulated (Table 1). The *cobA* gene, also known as *cysG* (Spencer et al., 1993; Woodcock et al., 1998), has been shown to be involved in sulfur and selenium metabolic pathways (Storbeck et al., 2009; Park et al., 2011; Moore et al., 2012). Accordingly, it was observed that several of the differentially expressed metabolites between *B. arboris* and the *ΔcobA* mutant contained these elements, indicating that *cobA* mutation affected the synthesis of the metabolites. The *cobA* gene also contributes to the biosynthesis of siroheme and vitamin B12 (Woodcock et al., 1998; Rodionov et al., 2003); however, the *ΔcobA* mutant did not show significantly reduced levels of heme and vitamin B12, which may be due to the detection sensitivity of the equipment or redundancy of *cobA* gene in *B. arboris*. Therefore, the antifungal activities of the three downregulated compounds in the *ΔcobA* mutant were evaluated.

**Table 1:** Metabolic differences between wild type *Burkholderia arboris* and ∆*cobA.*

Compounds	Formula	Class	Fold change	Mutant/WT	*p* value
Dimethyl diselenide	C_2_H_6_Se_2_	Heterocyclic compounds	0.371336	down	0.030657
LPE (0:0/16:0)	C_21_H_44_NO_7_P	GP	0.414760	down	0.031534
His-Ala-Phe-Lys	C_24_H_35_N_7_O_5_	Amino acid and its metabolites	0.479923	down	0.013086
Methylthiouracil	C_5_H_6_N_2_OS	Heterocyclic compounds	2.095310	up	0.037717
Mecoprop-P	C_10_H_11_ClO_3_	Benzene and substituted derivatives	2.388018	up	0.021607
Thiosulfate	HS_2_O_3_	Others	2.429764	up	0.02141
2-Mercaptobenzothiazole	C_7_H_5_NS_2_	Benzene and substituted derivatives	2.731294	up	0.02313
4-Hydroxy-2′,3,3′,5′,6′-pentachlorobiphenyl	C_12_H_5_Cl_5_O	Benzene and substituted derivatives	2.837713	up	0.034287
1-Hydroxy-2-naphthoic acid	C_11_H_8_O_3_	Benzene and substituted derivatives	2.854197	up	0.041737
Purine	C_5_H_4_N_4_	Nucleotide and its metabolites	2.896017	up	0.003918
2-O-(alpha-D-Mannosyl)-D-glycerate	C_9_H_16_O_9_	Organic acid and its derivatives	2.984335	up	0.029857
Halofuginone	C_16_H_17_BrClN_3_O_3_	Aldehyde, ketones, and esters	2.993056	up	0.022079
Resveratrol 4’-O-D-glucuronide	C_20_H_20_O_9_	Heterocyclic compounds	3.686397	up	0.031308
Deoxyribose 5-phosphate	C_5_H_11_O_7_P	Organic acid and its derivatives	4.034139	up	0.017055
Methyl 1-propene-1-sulfenoselenoate	C_4_H_8_SSe	Aldehyde, ketones, and esters	4.205002	up	0.002534
2.4-Dichlorobenzoic acid	C_7_H_4_Cl_2_O_2_	Benzene and substituted derivatives	4.995242	up	0.028888
1,4-Dichlorobenzene	C_6_H_4_Cl_2_	Benzene and substituted derivatives	5.972579	up	0.047527

Differential metabolites between wild type and mutant *B. arboris* were determined at variable importance in projection (VIP) ≥ 1, *p* < 0.05 (Student’s *t*-test), and absolute log_2_ fold change (FC) ≥ 1.0.

The three downregulated compounds were dimethyl diselenide, lysophosphatidylethanolamine (LPE or lysoPE), and His-Ala-Phe-Lys (Hafk peptide). Dimethyl diselenide is volatile with a low boiling point of 57°C at 760 mmHg (Lide, 2007). However, we found that *B. arboris* did not inhibit the growth of *F. oxysporum* in a petri dish with separate compartments, suggesting that the antifungal compounds are not volatile (Supplementary Figure 8). Similarly, 10 and 100 mM of LPE did not inhibit the growth of *F. oxysporum* on PDA media (Supplementary Figure 9), indicating that both dimethyl diselenide and LPE were not the antifungal compounds produced by *B. arboris*. Overall, these results indicated that Hafk peptide may be the compound with antifungal activity.

### Hafk peptide possesses antifungal activity

**Figure 5:**
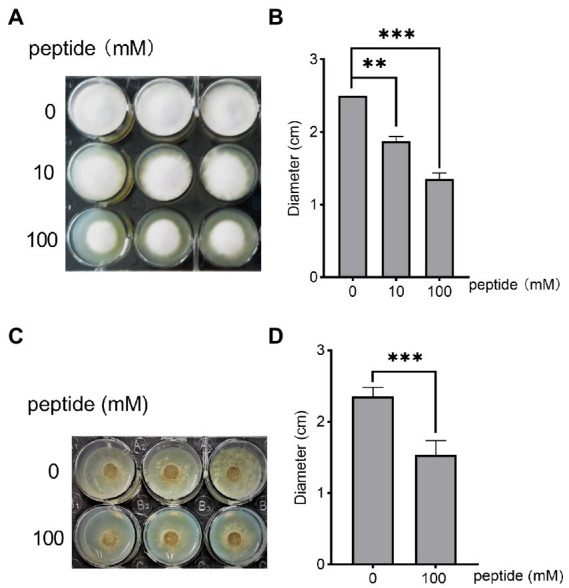
The peptide His-Ala-Phe-Lys (Hafk) antagonizes *Fusarium oxysporum* and *Botrytis cinerea*. **(A)** Growth inhibition assay of Hafk peptide against *F. oxysporum*. Different concentrations of Hafk peptide (50 μl) were evenly spread on the surface of the agar media in a 12-well-plate (diameter of each well, 25 mm), and then a fungal disk (diameter, 5 mm) was placed in the center of each well and cultured at 28°C. Images were taken 3 days after placement. A representative image is presented. **(B)** Hafk peptide inhibited the growth of *F. oxysporum*, as evidenced by the diameter of fungal mycelial growth. Data are presented as mean ± SD (*n* = 3). Asterisks indicate significant differences with or without the addition of the peptide (^**^p < 0.01, ^***^p < 0.001, Student’s *t*-test). The experiments were repeated with similar results. **(C)** Growth inhibition assay of Hafk peptide against *Botrytis cinerea*. The experimental conditions were the same as those in **(A)**, except that *B. cinerea* was used. **(D)** Hafk peptide inhibited the growth of *B. cinerea*, as evidenced by the diameter of fungal mycelial growth. Data are presented as mean ± SD (*n* = 3). Asterisks indicate significant differences with or without the addition of peptides (^***^p < 0.001, Student’s *t*-test). The experiments were repeated with similar results.

To investigate whether the short peptide Hafk possesses antifungal activity, we first determined the stability of crude extracts from *B. arboris* suspensions. The crude extracts were heated for 30 min at 40, 50, and 60°C respectively, followed by a plate confrontation experiment to verify the antifungal activity against *F. oxysporum* (Supplementary Figure 10 A). Preheating at high temperatures did not reduce the antifungal activity of *B. arboris*. Additionally, the crude extract exhibited antifungal activity after treatment with proteinase K, which catalyzes the degradation of L-isoforms of amino acids (Supplementary Figure 10 B), indicating that the antifungal compounds produced by *B. arboris* are not peptides or amino acids with L-isoforms (Kannan et al., 2020). Therefore, we synthesized a Hafk peptide containing all the D-amino acids, and its antifungal ability against *F. oxysporum* was assessed in a 12-well cell plate. PDA media (2 ml) was added at the bottom of each well, and then 50 μl of different concentrations of Hafk peptide solution was spread evenly on the surface. A *F. oxysporum* mycelium disk was placed at the center of each well and the mycelial growth zone was measured 72 h after placement. *Fusarium oxysporum* growth was significantly inhibited by the addition of 10 and 100 mM Hafk peptide compared with the control group; however, concentrations lower than 10 mM did not significantly inhibit fungal growth (Figures 5A,B). To detect whether Hafk peptide was secreted by *B. arboris*, the crude extracts from bacterial supernatant were subjected to LC–MS analysis. Synthesized Hafk peptide was used as a standard. The chromatogram in standard solution was overlapped with that in the supernatant of *B. arboris*, suggesting that Hafk peptide is present in the supernatant (Supplementary Figure 11). To test whether the Hafk peptide antagonizes other phytopathogenic fungi, its antifungal activity against *B. cinerea* was evaluated. Consistent with the findings in *F*. *oxysporum*, 10 mM of Hafk peptide significantly inhibited the growth of *B. cinerea* (Figures 5C,D). Similarly, *B. arboris* inhibited the growth of *B. cinerea* in the confrontation assay (Supplementary Figure 12). Overall, these results indicated that Hafk peptide possesses antifungal activity.

## Discussion

The identification of novel antifungal peptides has attracted much attention because it possesses considerable potential as alternatives to chemical pesticides or antibiotics in the control of fungal diseases in agriculture (Whetstone and Hammock, 2007; Fira et al., 2018; Zhao et al., 2019; Niu et al., 2020). In this study, a new short peptide (Hafk) consisting of four amino acids residues (His-Ala-Phe-Lys) was identified in *B. arboris*. The Hafk peptide with D-amino acids significantly inhibited the growth of *F. oxysporum* and *B. cinerea*, two phytopathogens that cause wilt and gray mold diseases. Overall, Hafk peptides could serve as a reference for the development of antifungal agents with broad-spectrum applications.

Antifungal peptides are produced by several organisms, including insects, plants, fungi, and bacteria (Maskey et al., 2003; Wen et al., 2014; Al Souhail et al., 2016; Khani et al., 2019). Most antifungal peptides are composed of 10–100 amino acid residues, and are classified as lipopeptides and linear, cyclic, and glucan peptides (Donzelli et al., 2001; Ortiz-Lopez et al., 2015; Park et al., 2018). Several mechanisms through which antifungal peptides inhibit fungal growth have been elucidated, which include the blocking of the biosynthesis of chitin and b-glucan, two major fungal cell wall components (Donzelli et al., 2001; Pushpanathan et al., 2012; Moghaddam et al., 2017; Tong et al., 2020). Additionally, some antifungal peptides can disrupt the fungal membrane, while others can pass through cell membranes to target intracellular DNA and RNA (Han et al., 2019; Khani et al., 2020; Bansal et al., 2022; Ding et al., 2022). Moreover, accumulating evidence indicates that short peptides containing 2–10 amino acids also exhibit antifungal activity (Velivelli et al., 2018; Gong et al., 2022). Compared to longer peptides, short antifungal peptides possess several advantages, including higher stability, lower toxicity to the host, and lower cost of synthesis (Apostolopoulos et al., 2021). However, the antifungal mechanisms underlying short antifungal peptides require further study. The Hafk peptide represents a new class of antifungal compounds; thus, it could serve as a model short peptide to elucidate the mechanism of antifungal activity.

Amino acids exist in two isomeric forms, D- and L-amino acids, with the exception of glycine (Du et al., 2019). Although L-amino acids are the most common form produced through chemical and enzymatic syntheses, D-amino acids are also involved in several biological processes. D-Ala and D-Glu were found to be the most common D-amino acids in bacterial extracellular or periplasmic polymers (Radkov and Moe, 2014). Several D-amino acids can synthesize biological peptides through the NRPS pathway, including antibiotics and toxins, such as daptomycin containing D-Ala (Baltz, 2009), which can inhibit spore growth (Shrestha et al., 2017). D-amino acids have also been reported to antagonize pathogens, such as *Agrobacterium tumefaciens*, *Mycobacterium smegmatis*, and *Saccharomyces cerevisiae* (Fox et al., 1944; Bopp, 1965; Yabu and Huempfner, 1974; Ruiz et al., 2021). In addition to their potential antimicrobial ability, D-amino acids are more stable than L- amino acids (Zhao et al., 2016). Therefore, D-amino acids could serve as experimental materials to elucidate the antimicrobial ability of peptides (Kapil and Sharma, 2021). In the present study, Hafk (His-Ala-Phe-Lys) peptide was identified in the wild-type using UPLC-QTOF/MS, while ∆*cobA* mutant showed reduced levels. Although ribosome *cobA* might not directly regulate the synthesis of D-amino acids, it is possible that ribosome *cobA* might control the production of a precursor substance which is required for the formation of mature Hafk peptide. Considering the cost, peptides with all 12 possible combinations of D and L isoforms were not synthesized, but instead assessed the antifungal activity of peptides with D-isoforms. In the present study, the Hafk peptide (D-isoform) significantly inhibited fungal growth; however, the concentration required is still high. Therefore, the antifungal activity of all combinations needs to be examined in future studies to improve the efficiency and reduce the cost.

CobA is homologous to the C-terminal module of CysG, which is a multiple functional enzyme involved in the metabolism of sulfur and selenium (Warren et al., 1994; Stroupe et al., 2003; Dereven'kov et al., 2017). In the present study, untargeted metabolomics between wild type and ∆*cobA* mutant identified compounds containing sulfur, selenium, and aromatic compounds. Since these elements are important for amino acid metabolism, it is possible that the *cobA* gene contributes to the biosynthesis of Hafk peptides. Moreover, *cobA* is important for the synthesis of cobalamin (vitamin B12) and siro heme (Storbeck et al., 2009; Park et al., 2011; Moore et al., 2012); however, both cobalamin and siroheme were not detected in the wild type and ∆*cobA* mutant, which could be attributed to low detection efficiency of the equipment or redundancy of *cobA* in *B. arboris*. Moreover, cobalamin and siroheme have not been reported to exhibit antifungal activity, indicating that both cobalamin and siro heme are not involved in antifungal activity in *B. arboris*. However, further studies are necessary to elucidate how *cobA* regulates the biosynthesis of Hafk peptide.

In summary, *B. arboris* exerts antifungal activity by producing Hafk peptide under the regulation of *cobA* gene. The Hafk peptide possesses considerable potential as a biocontrol agent for crop fungal diseases.

## Data availability statement

The raw data supporting the conclusions of this article will be made available by the authors, without undue reservation.

## Author contributions

HZ and CX conducted most of the experiments and analyzed the data. YC isolated *Burkholderia arboris* strain and initiated this project. HZ and YL wrote the manuscript. All authors contributed to the article and approved the submitted version.

## Funding

This work was supported by the Key Research and Development Program of Zhejiang Province (2021C02009, 2022C02016, and 2021C02064-7).

## Conflict of interest

The authors declare that the research was conducted in the absence of any commercial or financial relationships that could be construed as a potential conflict of interest.

## Publisher’s note

All claims expressed in this article are solely those of the authors and do not necessarily represent those of their affiliated organizations, or those of the publisher, the editors and the reviewers. Any product that may be evaluated in this article, or claim that may be made by its manufacturer, is not guaranteed or endorsed by the publisher.

## Footnotes

^1^ www.metware.cn
