# Supplementary material for: His-Ala-Phe-Lys peptide from Burkholderia arboris possesses antifungal activity
Source: Front Microbiol. 2022 Dec 6;13:1071530. doi: 10.3389/fmicb.2022.1071530 (PMC9763614; doi:10.3389/fmicb.2022.1071530)
Supplement: Supplementary file 1 [file Data_Sheet_1.docx]

Supplementary Material for

HAFK peptide from *Burkholderia arboris* possesses antifungal activity

Huajie Zhu^1^, Cuihong Xu^1^, Yicun Chen^2#^, Yan Liang^1#^

**Affiliation:**

^1^State Key Laboratory for Managing Biotic and Chemical Threats to the Quality and Safety of Agro-products, Institute of Biotechnology, Zhejiang University, Hangzhou 310058, China.

^2^Research Institute of Subtropical Forestry, Chinese Academy of Forestry, Hangzhou 311400, China

**^#^Correspondence:**

Yan Liang and Yicun Chen, Email: [yanliang@zju.edu.cn](mailto:yanliang@zju.edu.cn) and [yicun_chen@163.com](mailto:yicun_chen@163.com); Tel and Fax: 86-571-88982572 and 86-571-63327982

**Supplementary Figure 1.** The antibiotic sensitivity test of *Burkholderia arboris*.

**Supplementary Figure 2.** The plasmid map of pBBR1-mCherry.

**Supplementary Figure 3.** The plasmid map of pBBR1MCS-2.

**Supplementary Figure 4.** *Burkholderia arboris* possesses antifungal activity against *Fusarium oxysporum*.

**Supplementary Figure 5.** Tn5 transposon insertion mutants exhibit reduced antagonistic activities against *Fusarium oxysporum*.

**Supplementary Figure 6.** Detection of kanamycin resistant gene in M464.

**Supplemetary Figure 7.** Physiological characteristics of Δ*cobA* mutants.

**Supplementary Figure 8.** The antifungal compounds from *Burkholderia arboris* are not volatile.

**Supplementary Figure 9.** Lysophosphatidylethanolamine (LPE) has no antagonistic ability against *Fusarium oxysporum*.

**Supplementary Figure 10.** The antifungal compound from *Burkholderia arboris* was thermally stable and not sensitive to proteinase K.

**Supplementary Figure 11.** Representative multiple reaction monitoring (MRM) chromatograms of Hafk in bacterial supernatant.

**Supplementary Figure 12.** *Burkholderia arboris* possesses antifungal activity against *Botrytis cinerea*.

**Supplementary Table 1.** Strains and plasmids used in this study.

**Supplementary Table 2.** Primers used in this study.

**Supplementary Table 3.** Bacterial doubling and lagging time.

**Supplementary Table 4.** Raw data of untargeted metabolomic analysis using UPLC-QTOF-MS.

## Supplementary Figures


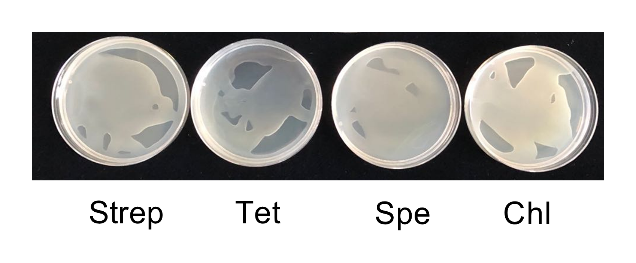


**Supplementary Figure 1.** The antibiotic sensitivity test of *Burkholderia arboris*. Streptomycin (Strep, 50 mg/mL), tetracycline (Tet, 15 mg/mL), spectinomycin (Spe, 50 mg/mL) and chloramphenicol (Chl, 50 mg/mL) were used to determine the antibiotic sensitivity of *B. arboris*. Bacterial suspension (100 μL) was spread on agar plates (60 mm) with the addition of the above indicated antibiotics, images were taken 1 d after incubation.


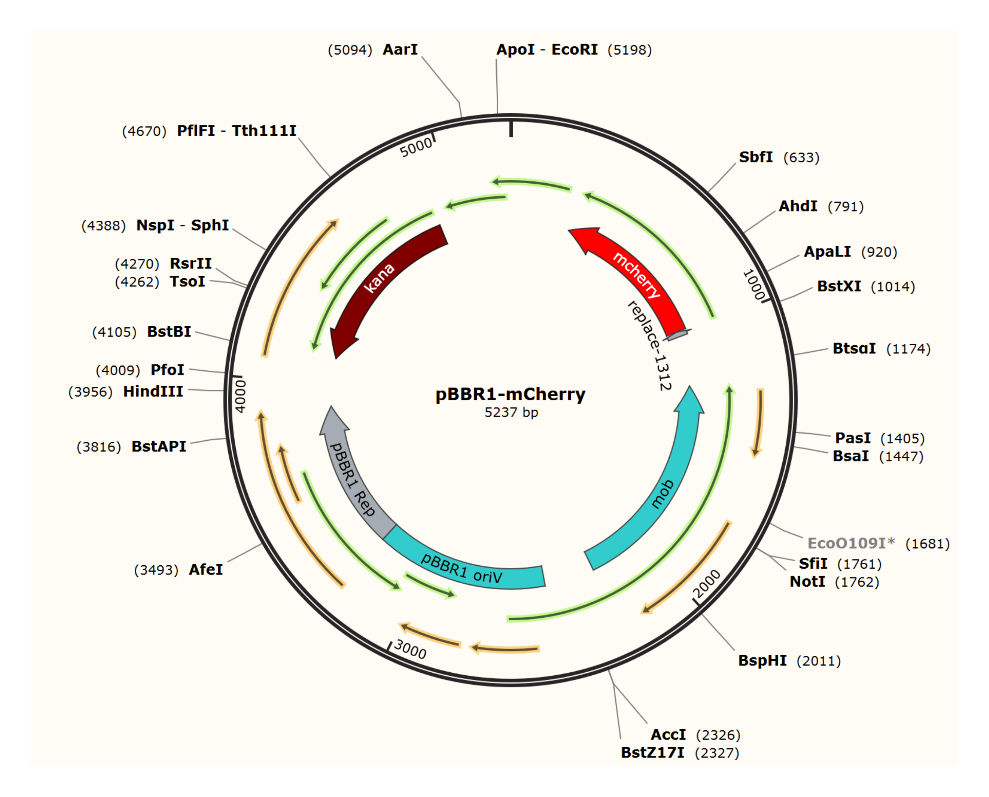


**Supplementary Figure 2.** The plasmid map of pBBR1-mCherry. Important elements include a kanamycin resistance gene (*Kan*), a *mob* gene, and an *mCherry* gene, are shown on the map.


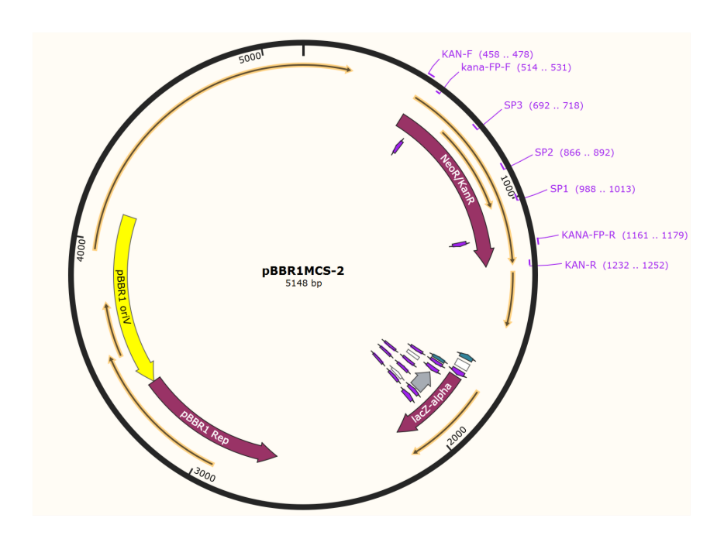


**Supplementary Figure 3.** The plasmid map of pBBR1MCS-2. Important elements include a *lacZ* gene and a kanamycin resistance gene (Kan), as shown on the map. The Kan gene fragment was amplified from pBBR1MCS-2 to construct a Tn5 transposon.


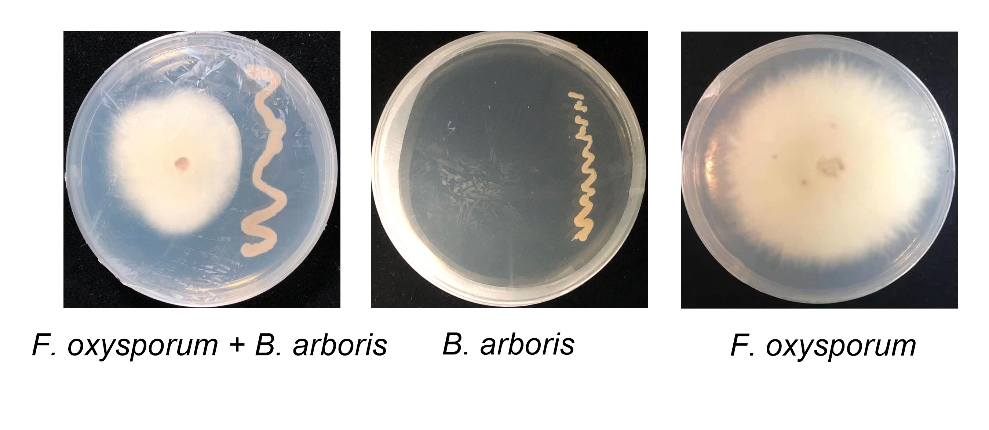


**Supplementary Figure 4.** *Burkholderia arboris* possesses antifungal activity against *Fusarium oxysporum*. The confrontation plate test of the antifungal activity of *B. arboris* against *F. oxysporum* was performed on PDA plates (90 mm), with *B. arboris* and *F. oxysporum* cultivated independently as controls. The experiments were repeated twice, and similar results were obtained.


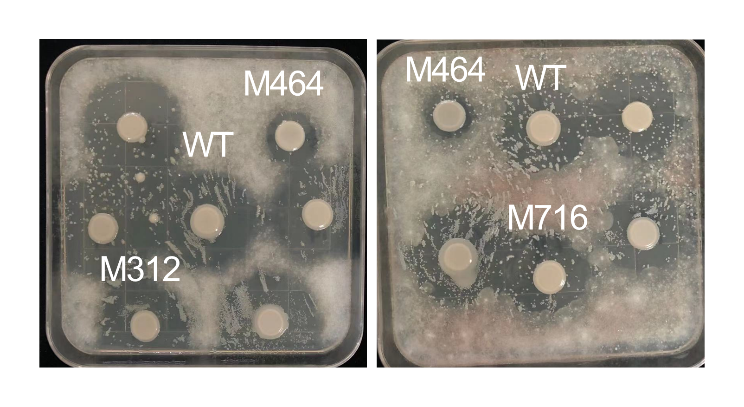


**Supplementary Figure 5.** Tn5 transposon insertion mutants exhibit reduced antagonistic activities against *Fusarium oxysporum*. M312, M464, M716 mutants showed reduced antifungal activities compared with the wild-type (WT), while M464 displayed the weakest antifungal ability.


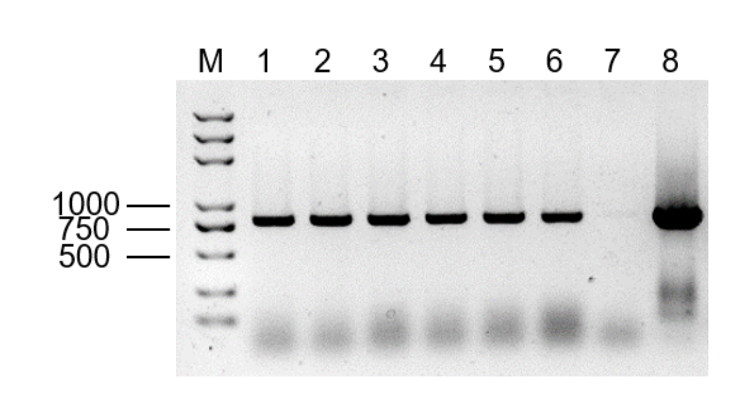


**Supplementary Figure 6.** Detection of kanamycin resistant gene in M464. The kanamycin resistant gene in wild-type (WT) *Burkholderia arboris* and M464 was amplified by PCR, and the PCR products were run on an agarose gel. The predicted product size was 833 base pairs (bp). PCR was run using different DNA as a template. Lanes 1- 6 were from M464, 7 was from WT *B. arboris*, 8 was the pBBR1MCS-2 plasmid, and M was the DNA marker.


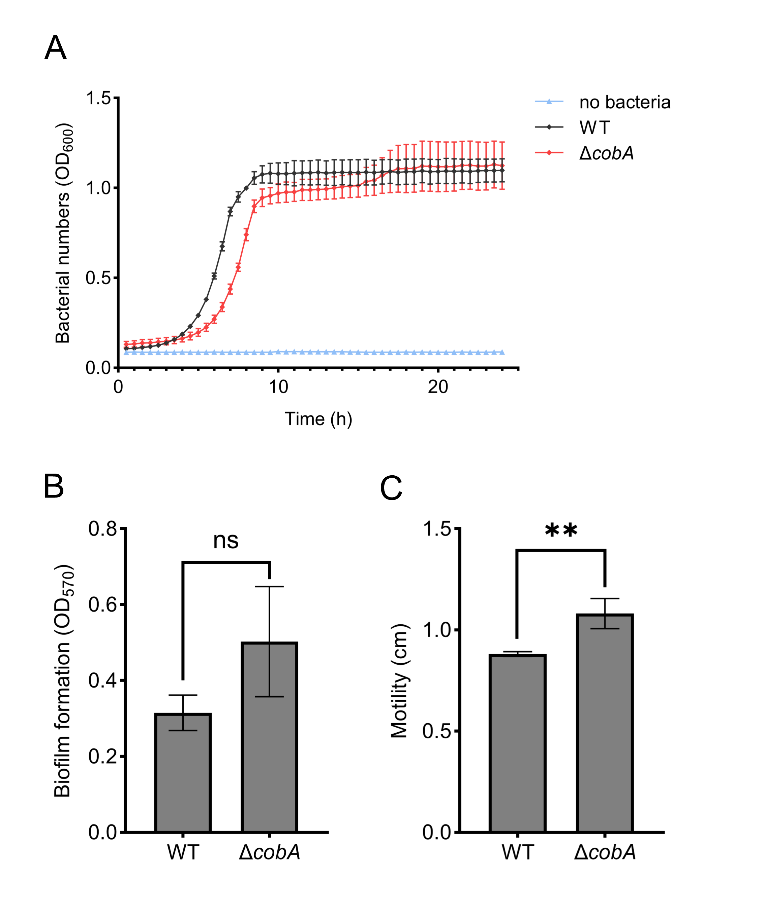


**Supplemetary Figure 7.** Physiological characteristics of Δ*cobA* mutants. **(A)** Growth curve. The bacterial growth rate of *Burkholderia arboris* wild-type (WT) and Δ*cobA* was determined by measuring the optical density at 600 nm. The growth curves were prepared from the growth of three independent colonies for each strain and the mean ± SD (n = 3) were plotted. **(B)** Biofilm formation. The biofilm formation of WT and Δ*cobA* was measured using a crystal violet assay. Data are presented as mean ± SD (n = 3). Ns indicates no significant difference between WT and Δ*cobA* (Student’s *t*-test). **(C)** Mobility. The mobility of the WT and Δ*cobA* was determined by measuring the diameter of the swimming area. Data are presented as mean ± SD (n = 3). Asterisks indicate significant differences between the WT and Δ*cobA* mutants (***p* < 0.01, Student’s *t*-test). The experiment was repeated with similar results.


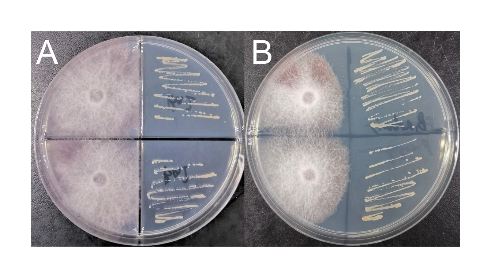


**Supplementary Figure 8.** The antifungal compounds from *Burkholderia arboris* are not volatile. **(A)** Confrontation plate test of the antifungal activity of *B. arboris* against *F*. *oxysporum* was performed in a petri dish (90 mm) with separated compartments. **(B)** Confrontation plate test in a petri dish (90 mm) without a separate compartment. The streaked *B. arboris* bacteria and *F. oxysporum* disks (diameter, 0.5 cm) were co-cultivated on the PDA plates. Images were taken 6 d after cultivation.


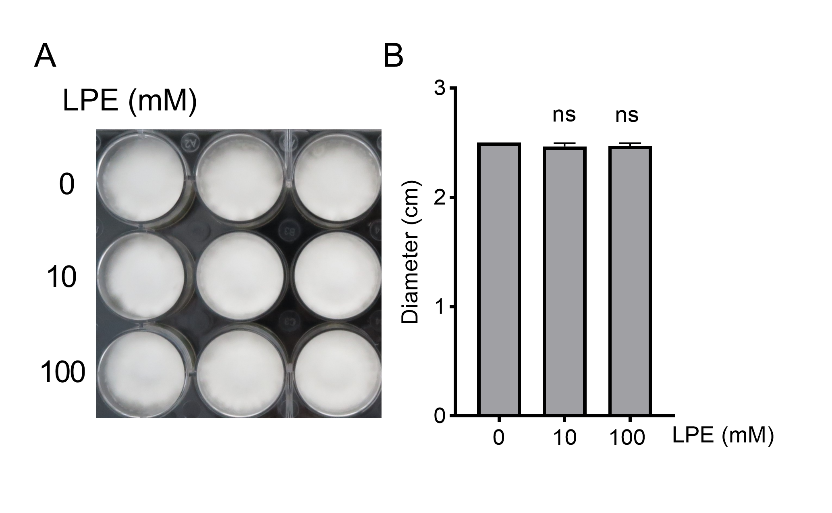


**Supplementary Figure 9.** Lysophosphatidylethanolamine (LPE) has no antagonistic ability against *Fusarium oxysporum*. **(A)** Image of *F. oxysporum* growth. Different concentrations of LPE (50 μL) were evenly spread on the surface of PDA media in each well (25 mm diameter), with *F. oxysporum* disks (5 mm diameter) placed on the top of the surface. Images were taken 3 d after cultivation. **(B)** Diameter of mycelial growth of *F. oxysporum*. Data are presented as means ± SD (n = 3). Ns indicates no significant differences between treatments and control (Student’s *t*-test).


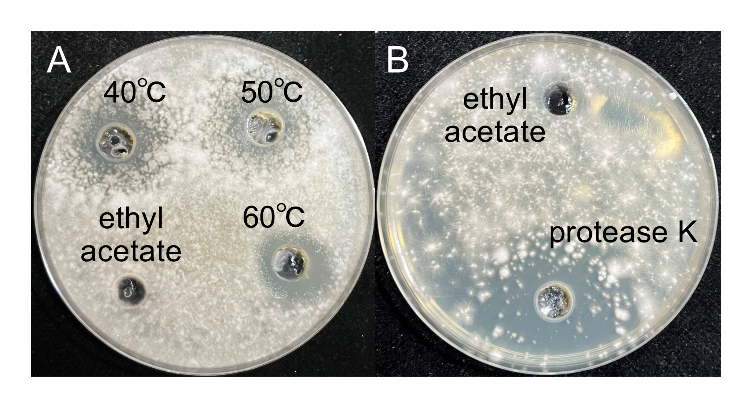


**Supplementary Figure 10.** The antifungal compound from *Burkholderia arboris* was thermally stable and not sensitive to proteinase K. **(A)** The antifungal compound isolated from *B. arboris* was thermally stable. Crude metabolic extracts from *B. arboris* were heated at 40, 50, or 60 ℃ for 0.5 h, and their antifungal activities against *Fusarium oxysporum* were determined on PDA plates (90 mm). Ethyl acetate was used as a negative control. The experiments were repeated twice, and similar results were obtained. **(B)** The antifungal compound from *B. arboris* was not sensitive to proteinase K. Crude metabolic extract from *B. arboris* was treated with proteinase K (20 mg/mL) for 0.5 h, and the antifungal activities against *F. oxysporum* was determined on PDA plates (90 mm). The experiments were repeated twice, and similar results were obtained.


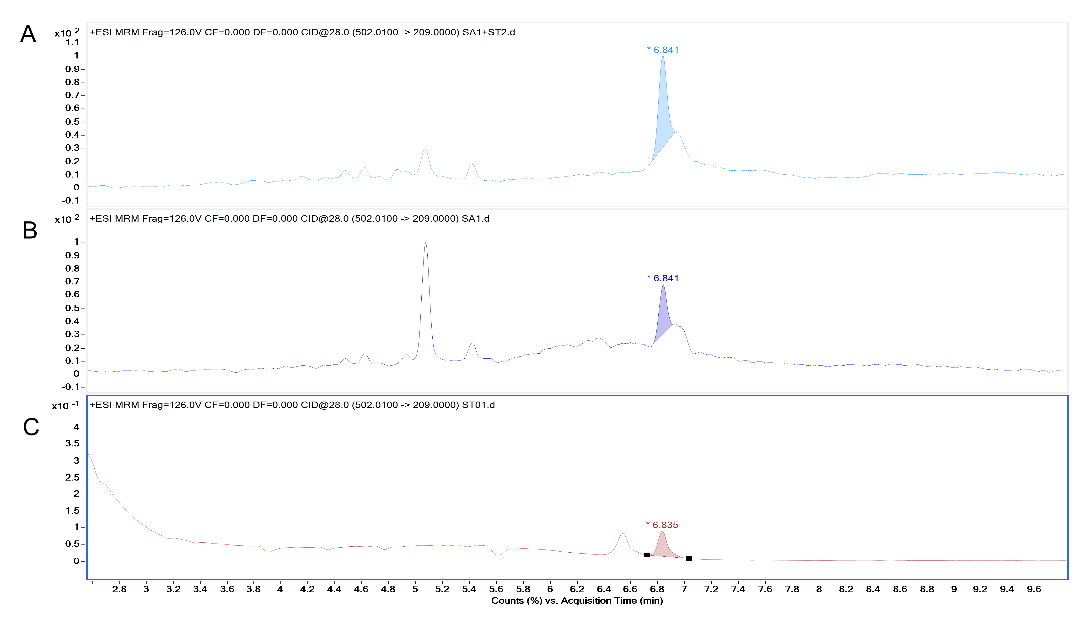


**Supplementary Figure 11.** Representative multiple reaction monitoring (MRM) chromatograms of Hafk in bacterial supernatant. (A) Bacterial supernatant sample spiked with Hafk standard sample. (B) Bacterial supernatant sample. (C) Hafk standard sample.


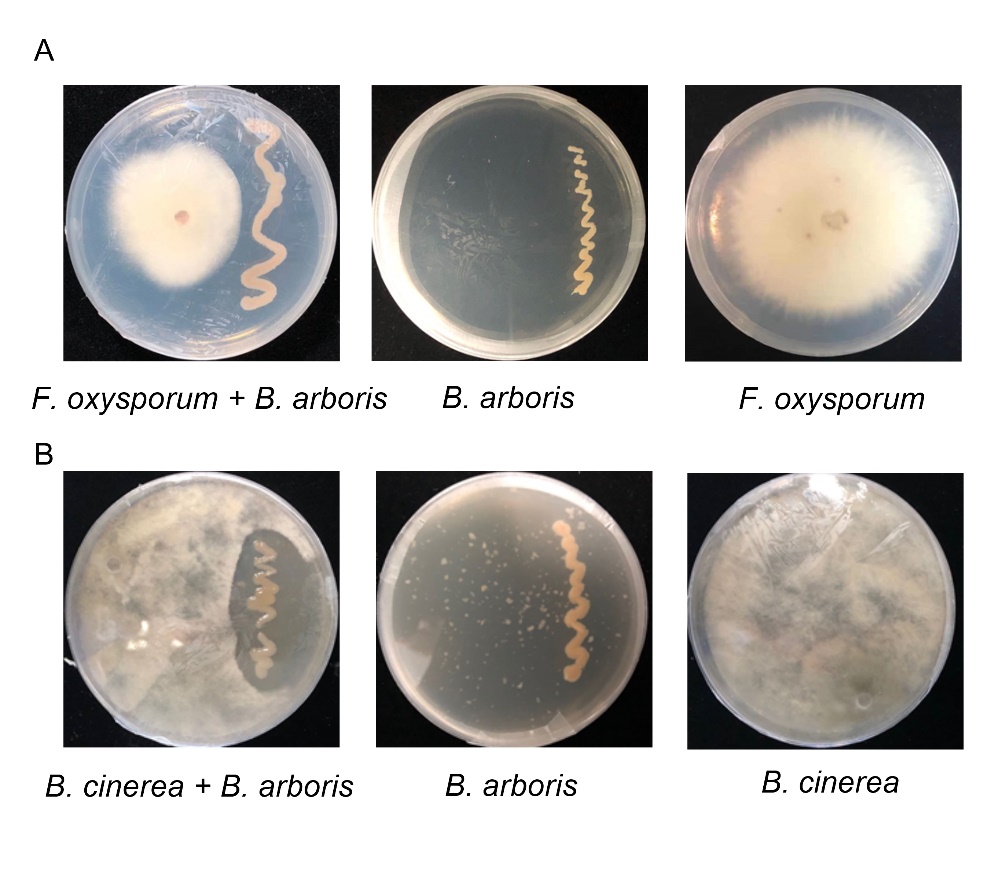


**Supplementary Figure 12.** *Burkholderia arboris* possesses antifungal activity against *Botrytis cinerea*. The confrontation plate test of the antifungal activity of *B. arboris* against *B. cinerea* was performed on PDA plates (90 mm), with *B. arboris* and *F. oxysporum* cultivated independently as controls. The experiments were repeated twice, and similar results were obtained.

**Supplementary Table 1. Strains and plasmids used in this study**

| **Strains or plasmids** | **Description** | **Source or reference** |
| --- | --- | --- |
| **Strains of *Burkholderia arboris*** | | |
| *Burkholderia arboris 1* | Wild type | CGMCC No.16905 |
| *B. arboris*-mCherry | Fluorescent labeled strain, Km^R^ | This study |
| M 464 | A Tn5 inserted mutant, Km^R^ | This study |
| Δ*cobA* | A knockdown mutant, Km^R^ | This study |
| **Strains of *Escherichia coli*** | | |
| DH5α | Recombinant plasmid replication | Laboratory stock |
| S17-1 λpir | Recombinant plasmid replication | Laboratory stock |
| **Other fungal strains** | | |
| *Fusarium oxysporum* f. sp. *lycopersici* |  | Laboratory stock |
| *Botrytis cinerea* B05.10 |  | Laboratory stock |
| **Plasmids** | | |
| pJP5603 | Suicide plasmid, Km^R^ | Laboratory stock  (Riedel et al., 2013) |
| pJP5603-*cobA* | pJP5603 with *cobA* flanking sequences, Km^R^ | This study |
| pBRM | Km^R^, *mCherry* | Laboratory stock  (Kovach et al., 1995) |
| pCE2 | Recombinant plasmid replication, Km^R^ | Laboratory stock |
| pCE2-MEKANA | For EZ-Tn5 transposome | This study |

**Supplementary Table 2. Primers used in this study.**

Underlined sequences and contents in parentheses represent restriction enzyme sites.

| Primer | Primer sequences (5’→3’) | Product length (bp) | |
| --- | --- | --- | --- |
| 16S-F | CTTGGCCCTAATACGGTCGGG | | 564 |
| 16S-R | CCACCTCTCAGCGGGATTCCGA | |  |
| *mCherry*-F | TTACTTGTACAGTTCGTCCATGCCG | | 711 |
| *mCherry*-R | ATGGTGAGCAAGGGCGAGGAGG | |  |
| pJP5603-mcs-F | GCAATTAATGTGAGTTAGCTCACTC | | - |
| pJP5603-mcs-R | GCCATTCAGGCTGCGCAACTG | |  |
| qrt16S-F | CCTACCAAGGCGACGATCA | | 103 |
| qrt16S-R | CAAAATTCCCCACTGCTGCC | |  |
| MEKANA-F | CTGTCTCTTATACACATCTATGATTGAACAAGATGGATTG | | 833 |
| MEKANA-R | CTGTCTCTTATACACATCTTCAGAAGAACTCGTCAAGAAG | |  |
| AD1 | NTCGASTWTSGWGTT | | - |
| AD2 | NGTCGASWGANAWGAA | | - |
| AD3 | WGTGNAGWANCANAGA | | - |
| AD4 | TGWGNAGWANCASAGA | | - |
| AD5 | AGWGNAGWANCAWAGG | | - |
| AD6 | CAWCGICNGAIASGAA | | - |
| KANA-SP1 | CGACGAGATCCTCGCCGTCGGGCATG | | - |
| KANA-SP2 | ACGTGCTCGCTCGATGCGATGTTTCGC | | - |
| KANA-SP3 | CGCTTCAGTGACAACGTCGAGCACAGC | | - |
| *cobA*-F | CGCGGATCCCCATGGGCAAGGTGTATCTGAT (*Bam*HI) | | 744 |
| *cobA*-R | CGGAATTCGGCCGCATTTCGCAGCGAAT (*Eco*RI) | |  |

**Supplementary Table 3. Bacterial doubling and lagging time.**

| Strains | Doubling Time (h) | Lagging Time (h) |
| --- | --- | --- |
| Wild type | 6.94 | 9.00 |
| Δ*cobA* | 7.73 | 9.50 |

**References**

Kovach, M.E., Elzer, P.H., Hill, D.S., Robertson, G.T., Farris, M.A., Roop, R.M., 2nd, et al. (1995). Four new derivatives of the broad-host-range cloning vector pBBR1MCS, carrying different antibiotic-resistance cassettes. *Gene* 166**,** 175-176.

Riedel, T., Rohlfs, M., Buchholz, I., Wagner-Dobler, I., and Reck, M. (2013). Complete sequence of the suicide vector pJP5603. *Plasmid* 69**,** 104-107.
